# Lessons learned from implementing a responsive quality assessment of clinical ethics support

**DOI:** 10.1186/s12910-019-0418-2

**Published:** 2019-11-01

**Authors:** Eva M. van Baarle, Marieke C. Potma, Maria E. C. van Hoek, Laura A. Hartman, Bert A. C. Molewijk, Jelle L. P. van Gurp

**Affiliations:** 1 Netherlands Defense Academy, Breda, the Netherlands; 2 Amsterdam UMC, VU University Medical Centre (VUmc), Amsterdam, the Netherlands; 3 Department of Care Ethics, University of Humanistic Studies (UvH), Utrecht, the Netherlands; 4 Amsterdam UMC, VU University Medical Centre (VUmc), EMGO+, Amsterdam, the Netherlands; 5 Centre for Medical Ethics at the University of Oslo (UIO), Oslo, Norway; 6 IQ Healthcare Department, Radboud University Medical Center Nijmegen, Nijmegen, the Netherlands

**Keywords:** Responsive evaluation, Responsive quality assessment, Clinical ethics support, Learning network

## Abstract

**Background:**

Various forms of Clinical Ethics Support (CES) have been developed in health care organizations. Over the past years, increasing attention has been paid to the question of how to foster the quality of ethics support. In the Netherlands, a CES quality assessment project based on a responsive evaluation design has been implemented. CES practitioners themselves reflected upon the quality of ethics support within each other’s health care organizations. This study presents a qualitative evaluation of this Responsive Quality Assessment (RQA) project.

**Methods:**

CES practitioners’ experiences with and perspectives on the RQA project were collected by means of ten semi-structured interviews. Both the data collection and the qualitative data analysis followed a stepwise approach, including continuous peer review and careful documentation of the decisions.

**Results:**

The main findings illustrate the relevance of the RQA with regard to fostering the quality of CES by connecting to context specific issues, such as gaining support from upper management and to solidify CES services within health care organizations. Based on their participation in the RQA, CES practitioners perceived a number of changes regarding CES in Dutch health care organizations after the RQA: acknowledgement of the relevance of CES for the quality of care; CES practices being more formalized; inspiration for developing new CES-related activities and more self-reflection on existing CES practices.

**Conclusions:**

The evaluation of the RQA shows that this method facilitates an open learning process by actively involving CES practitioners and their concrete practices. Lessons learned include that “servant leadership” and more intensive guidance of RQA participants may help to further enhance both the critical dimension and the learning process within RQA.

## Background

CES in healthcare organizations is regarded a key service to support health care professionals in reflecting on and fostering the quality of care. Therefore, a range of CES services have been developed within health care organizations [1–7] CES includes ethics committees [3, 8, 9] ethics consultation [10], moral case deliberation [6, 7], moral counselling [11], and various forms of ethics education [12]. The common denominator in these CES activities is a focus on fostering ethical reflection and well-considered decision making.

As CES can be a ‘high-stakes endeavour that influences clinical practice in critical aspects of health care’ [13], attention to both guaranteeing and fostering the quality of CES has increased. In particular, scholars have paid attention to the quality of CES in terms of richness of the content and quality of argumentation [14, 15] and to the quality and competences of its practitioners [13, 16–20]. Drawing parallels to standards required in other clinical disciplines, the need for professionalization and standardization within the domain of CES has been emphasized by Dubler and Blustein [21]. As a consequence, an increasing awareness of the importance of research on the quality of CES has resulted in some initial approaches to describing the quality of CES. For instance, to promote accountability in the quality of ethics consultation, an Ethics Consultation Quality Assessment Tool (ECQAT) was developed. ECQAT enables individuals who rate the quality of ethics consultation to make their assessments based on the written record [22, 23].

Focusing on the quality of CES practitioners themselves, a number of studies have found that a low percentage of clinical ethics committee (CEC) members are ‘ethics specialists’. Short training programmes for CES practitioners are said to litter the field [16]. An approach to assessing the quality of CES practitioners is the ‘Code of Ethics and Professional Responsibilities for Healthcare Ethics Consultants’ from the American Society for Bioethics and Humanities (ASBH) [20]. This society stresses that a clear definition of what a clinical ethicist is remains elusive. Therefore, this code is viewed as a living document based on a participatory process used to develop a code of practice standards for CES practitioners [20]. In Canada, there is a similar initiative to develop practice standards for ‘practising healthcare ethicists exploring professionalization’. These practice standards aim to prompt CES practitioners to reflect on the importance of context, process and principles, not just outcomes, in the exploration of and possible movement towards professionalization [24].

Supported by the Dutch Ministry of Health, Welfare and Sport, a Dutch national learning network for ethics support (NEON) was established in 2013 [25, 26]. NEON aims to bring CES practitioners together to learn from each other’s experiences, professionalize CES by stimulating knowledge exchange and reflect on the quality of CES activities. To reach these aims, NEON has organized national CES conferences, published a Dutch CES handbook [26], organized specific CES workshops and developed a website and publishes a regular CES newsletter.

In addition, NEON organized a reflective quality assessment in and together with 11 Dutch health care organizations in 2017–18. The point of departure of this quality assessment project is based on the idea that the quality of CES cannot be defined beforehand but should be understood as an ongoing reflective dialogue in the specific contexts of CES practitioners [25]. In other words, the RQA was conducted by CES practitioners who, by visiting other CES practices and exchanging CES experiences with colleagues, co-created an open learning process in which both the quality of CES and how to foster the quality of CES were reflected upon. This RQA project resembles the approach described by Tarzian and colleagues, as it is focused on a process of developing quality criteria in interaction with professionals who are active in CES. However, in contrast to the ASBH, the Dutch network addresses a larger group of stakeholders, and they are given a more active role in the learning process of fostering the quality of CES [23, 27].

The aim of this study is to evaluate the abovementioned RQA of CES in Dutch health care organizations, together with the RQA participants. We address the following research questions**:** 1) What are the experiences with and lessons learned from RQA on CES in Dutch health care organizations? and 2) What is the perceived value of the method of RQA for reflecting on the quality of CES?

## Methods

We used a qualitative research approach to explore experiences with and lessons learned from responsive quality assessments of CES in Dutch health care organizations. This qualitative approach allowed us to gather and analyse experiences and learning processes. The empirical data will form the basis for a critical appraisal of RQA as well as for developing andimproving this particular way of evaluating the quality of CES.

### The responsive quality assessment (RQA) project

The overall objective of the project was to both reflect upon and foster the quality of CES in Dutch health care organizations through a mutual learning process. The assessment was based on a responsive evaluation design in which CES practitioners themselves reflected upon the quality of ethics support within another health care organization.

The project originally consisted of 11 participating health care organizations. Due to withdrawal from the project, 10 health care organizations completed the RQA. These health care organizations represented different health care contexts. The project included four institutions for people with disabilities, two institutions for people with mental problems or psychiatric disorders, two academic hospitals, and two secondary hospitals.

**Fig. 1:**
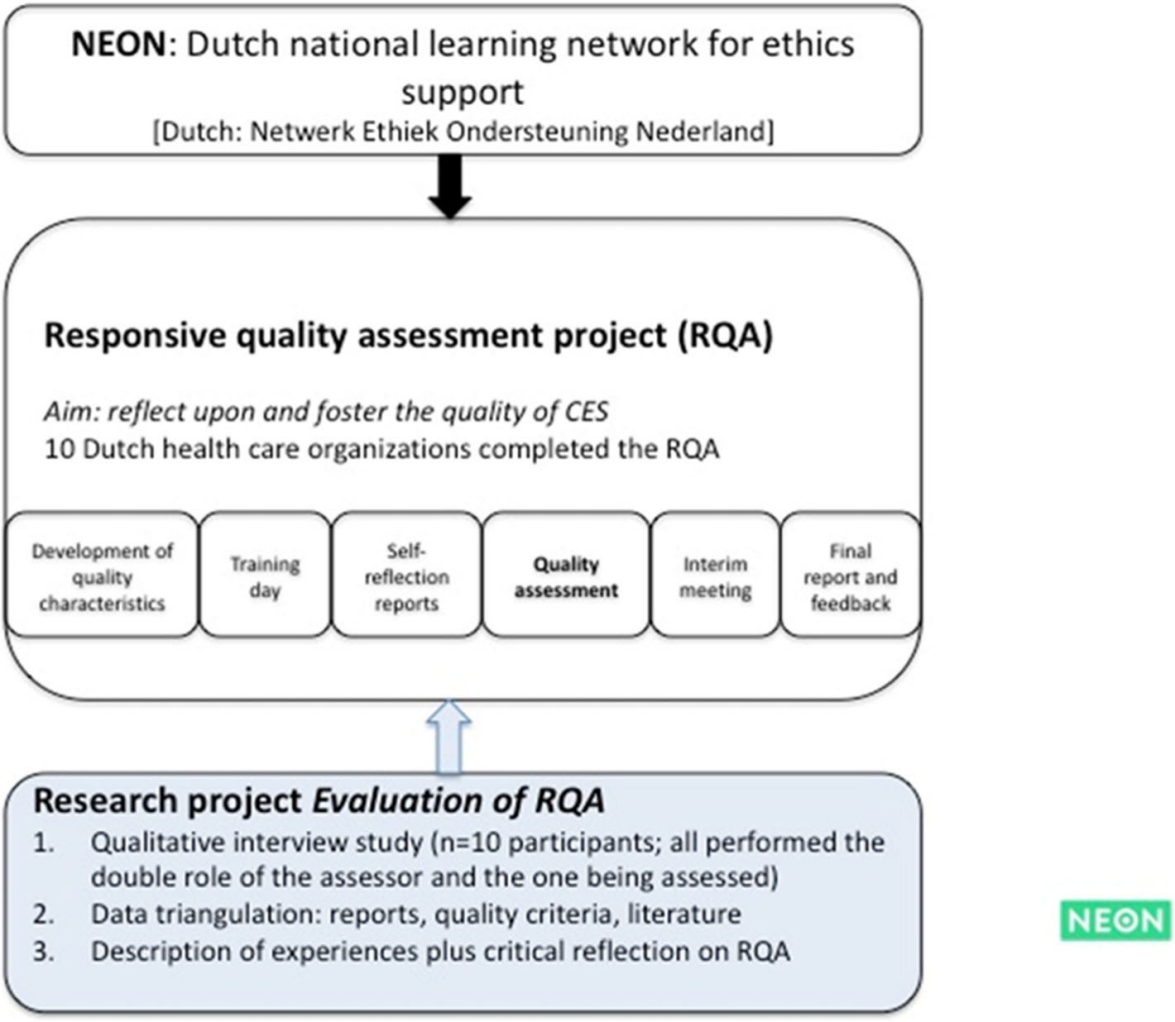
The RQA project

In the build-up to the RQA, a set of quality characteristics was developed in interaction with professionals who are active in CES [26]. These quality characteristics included characteristics regarding the goals and content of CES activities, different CES activities, competencies of CES practitioners and the implementation of CES services. The quality characteristics were not presented as norms or rules by which to judge the CES activities in the participating organizations but rather as a heuristic starting point for further reflection. Then, a call was posted on the NEON website for Dutch CES practitioners in Dutch health care organizations to participate in the RQA project. From each of the participating organizations, two CES practitioners participated in this assessment project. Each participating CES practitioner had a double role: hosting other CES practitioners for a quality assessment and executing a quality assessment him/herself in another health care organization. First, 22 CES practitioners had a training day to build relationships among CES practitioners and to receive training on how to responsively evaluate each other’s practices (see Fig. 1). To begin the assessment, CES practitioners collected information on CES services within their own organization. In addition, they were asked to write a reflection report on CES services within their own organization. All information was sent to the CES practitioners visiting their organization. Subsequently, pairs of CES practitioners from two different organizations would visit a third organization where they would assess the quality of CES. When approximately one-third of the visits had taken place, an interim meeting was organized by the research team to reflect on the process, 10 participants attended in this meeting. Following their visit, visiting CES practitioners wrote a final report that was discussed with the hosting CES practitioners during a feedback meeting or by email.

### Data collection

The experiences and perspectives of ten CES practitioners regarding the RQA project were collected by means of semi-structured qualitative interviews conducted by three experienced qualitative interviewers. Inspired by the idea of “Grand Tour Questions” [28], we designed a topic guide, including questions for every topic that invited participants to guide the interviewers through their personal quality assessment trajectory, thereby reflecting on the process as well as the content of the quality assessments. In addition, prior quality assessment reports, the aforementioned quality characteristics, and themes from earlier publications on the NEON learning network [26] provided us with particular topics for discussion during the interviews. The topic guide was developed and refined in several research group discussions before we conducted the interviews, thereby guaranteeing reliability.

In addition to the interviews, the RQA project provided data that could be used for data triangulation and more accurate interpretation [29]. Data that could be used in this way included the minutes from the training session and feedback meetings, the reflection reports, and the quality assessment reports. See Table 1 for the empirical data used in this paper.

**Table 1:** Empirical data

Data types	Use in analysis
1. 10 semi-structured interviews (duration 60–90 min)	Primary data for thematic analysis
2. NEON Handbook; quality characteristics for CES	Development of the topic guide
3. Final quality assessment reports	Development of the topic guide; data triangulation
4. Minutes of the training session; interim meeting and feedback meeting	Data triangulation
5. Self-reflection reports on CES services within their own organization	Data triangulation

### Data analysis

A team of researchers with different roles in the RQA project (assessor; project management; independent researcher) and various professional backgrounds (philosophy; medical ethics; military ethics; ethics of care; social science; biomedical sciences) conducted a collaborative inductive thematic analysis [30, 31]. Starting out with the interview data, the team aimed for “identifying, analysing, and reporting patterns (themes and subthemes)” [30]. Initially, the analysis focused on recognizing and mapping patterns in the experiences of the interviewees with the RQA. Starting with the first interview, the research team aimed to inductively build an overview of these patterns in interviewees’ experiences through open coding. Using each new interview to check and validate already recognized themes, and add new themes as they emerged, a saturated description of themes was the final result. Second, in the process of continuously validating themes the team of researchers was able to demonstrate connections between themes and to distinguish themes (of a higher level) from subthemes. Some clusters of subthemes had no distinct label stemming from the raw data and were therefore interpreted and denominated by the researchers [30, 32]. The result of this second analytical step was a presentation of logically connected themes, subthemes, and quotations (raw data) [30, 32]. Through this collaborative approach, the researchers were able to be systematically critical of one another’s interpretations and reflective on one’s personal predispositions [31].

The analysis followed a stepwise approach:

Step 0: the audio recordings of the interviews were transcribed ad verbatim.

Step 1: four data analysts (EvB, MP, MvH, and JvG) independently coded the first interview transcript inductively. No pre-existing coding frame was available, but analyses were guided by sensitizing concepts such as ‘responsive evaluation’, ‘impact/effect of the quality assessment project’, ‘critical reflection’ and ‘awareness of quality of CES’. A collaborative discussion followed the open coding, resulting in a tentative overview of themes. The codes as well as the overview were recorded in Caqdas F4, the peer review discussion and accompanying decisions recorded in memos.

Step 2: the remaining nine interviews were again coded independently and then critically discussed in pairs. In four collaborative discussions, the tentative overview of themes was further specified. This refinement procedure was also recorded in F4. The independent coding of the last interviews showed strong congruence between coders, hinting at strong reliability of the coding process. In this phase, the data analysis group also discussed a first distinguishing between themes and subthemes using constant comparison. The research group, including the other co-authors (LH and BM), used peer review to further validate the overview of themes and subthemes.

Step 3: in a final collaborative session, the categorization into themes was finalized and again presented in the research group. When clusters of subthemes lacked a common denominator, the researchers interpreted and labelled these clusters.

Step 4: the researchers performed a critical reading of the secondary data (data triangulation) to validate and potentially further specify the emergent themes. Any changes in this stage of the analysis were discussed by the research group and recorded in memos.

### Findings

In this section, we present the main findings with regard to how participating CES practitioners perceived the quality assessments and its yield (see also Table 2). We present 1) motives for participating, 2) perceived changes regarding CES as a result of the RQA, 3) the need for a learning community on CES, and 4) the perceived quality of the RQA. In the Discussion section, we highlight the implications of these findings for future RQAs to further develop and foster the quality of CES.

**Table 2:** Overview of themes and subthemes

Themes	Subthemes	Quotation
Motivation	- learning from each other’s CES practices - Curiosity - Strategy	*My objective was simply to see how other health care organizations organize CES and to learn something I could really use in my own organization* (R8)
Perception of the objective of the assessment	- Audit or assessment - Score list or quality characteristics - Professionalization	*A score list doesn‘t do justice to what happens there in practice (…) Implementation is a word we no longer use here, or unroll, that word is even worse (…). We focus much more on how we can stimulate people, tempt them to start new things (…) By connecting with the things people are intrinsically motivated to do* (R6)
Preparation of the assessment	- Preparing questions - Attitude (open or critical) - Strategy - Division of work	*Within the organization we visited, the CES practitioners decided to be present during all the conversations we had. This was a deliberate choice. While, on the other hand, we deliberately decided not to be present during the entire assessment* (R2)
Perceived changes as a result of the quality assessment	- New CES methods - Acknowledgement of CES - Formalizing CES practices - Inspiration - Self-reflection	*They had a yearly meeting. But really well designed. On the Internet, and also brochures and the like. With videos, so we would really indulge. Incredibly professional. I thought, goodness, this is fantastic* (R7)
Context of CES in the organization	- Workload - Organizational changes - Place of CES in organization	*You can participate in the national training [moral case deliberation] which is very interesting but also very expensive. Well, it would be nice if this could be facilitated as it will result in better skilled employees [CES practitioners]* (R6)
NEON as a learning community	- Schooling - Support/ Being part of a national CES community - Professionalization	*And I very specifically would like to get some expertise in the field of healthcare ethics (…) I am thinking about some kind of ethical deliberations (…) a sort of basic training in ethics* (R7)
Who is the assessor	- Background/ education - Competences - Feelings regarding the assessment	*I feel quite competent as an assessor. But I do not feel like an ethicist. And, er, that also has to do with the fact that I [silence] have not followed a specific training for it* (R7)
Quality of the assessment	- Interaction between respondents (as assessors) - Workload - CES knowledge/ competencies of the assessor	*So yes, there was a conflict, there were conflicting signals. This is confidential isn’t it. It is just a small example. So how you do work with each other there were actually, er, conflicting signals in our interactions* (R4)

#### Motivations for participating in the RQA

Two main motives for participating in the RQA were identified. The first was to learn from each other’s CES practices and the second, to think about improving the strategic position of CES services in their own particular health care organizations.

##### Learning from each other’s CES practices

All respondents were enthusiastic about participating in the RQA project. Their main motivation was to learn from each other and to obtain practical suggestions to improve their own CES practices.

Respondents were motivated to participate in the quality assessment project since, from the beginning, the assessment was introduced by the research team as an open learning process rather than an audit. Most respondents disliked the idea of an audit, which they associated with having to comply with a predefined normative framework that does not necessarily fit their CES practices.

*And the idea of looking in a critically constructive way into how we organize that [… ] and how we [as CES practitioners] can be meaningful within the organization. That truly spoke to us. […] We are a rather open and open-minded organization, and you [interviewer] were also very clearly the same. We do not let some sort of committee assess us; in fact what we really want is to examine together in practice the current position of organizations, care organizations, and how we could say something about that in terms of quality. That was appealing. And so there are two reasons: intrinsically speaking in terms of where we are now as an organization, and also the way in which it is presented – it's a match. (R10)*

##### Strategic reasons for participating

Respondents observed that CES often lacks a formal status within organizations as well as a substantial budget. The analysis showed that such a context could influence the motivations of participating CES practitioners. Respondents mentioned a strategic motivation for participating in the RQA to increase awareness of CES in their own health care organizations, more specifically within their board of directors.

*The external evaluation can cause you to be taken more seriously internally. With such a positive report, it is easier for us to go back to the board of directors […] and it gives you leverage to ask for more. (R1)*

Apart from the budgetary concerns, fragmentation of CES services is common, and a clear definition of responsibilities is usually absent.

*We just do not have an ethics committee, we do not have any people who formally work with ethics, not even informally. (R9)*

#### Perceived changes regarding CES due to the RQA

Due merely to participating in the RQA, respondents perceived various concrete changes with regard to CES services within their organization. This includes an increased awareness of the relevance of CES and of formalizing CES activities within their organizations. Furthermore, the assessments offered inspiration for developing new CES-related activities and increased self-reflection regarding existing CES activities within participating organizations.

##### Acknowledgement of the relevance of CES

The interviews show that it is important for CES practitioners that their work is acknowledged as relevant for the quality of care. According to the respondents, this acknowledgement is still lacking in some of the participating organizations. The RQA contributed to the acknowledgement of the relevance of CES in various ways. For instance by considering financial compensation for CES practitioners:

*Some people were able to do it [CES] more or less as part of their position and were compensated for it via their association, but others were not. We were startled and spoke out about it because you‘re asking quite a lot from CES practitioners, learning about moral deliberations in their own time – and then not being compensated. He [the director] first gave a rather business-like answer and later also realized that, yes, that may be a topic which may need to be revisited. (R7)*

*The acknowledgement of CES also included introducing new ways to organize CES services within the organization:*

*The consequences of participating in the quality assessment is that we are taken more seriously and we are facilitated […] Moral deliberations may now be easier to request. And it is a fact that a budget has now been allocated for moral deliberations. (R1)*

##### Formalizing CES practices

As mentioned above, in several health care organizations, there is a lack of a formal role for CES. In a number of organizations, however, participating in the quality assessments project resulted in direct financial support for CES.

*I also know that that [participating in the RQA] has worked. Policy on this issue does change. Those people [CES practitioners] now do get the financial compensation. (R7)*

##### Inspiration for developing new CES-related activities

The quality assessments inspired participating CES practitioners to use different and more diverse methods and activities with regard to CES in their organizations. The different methods of ethics support also inspired practitioners to broaden their views on ethics and CES in general. Ethics came to be understood as more than simply applying one specific method, such as moral case deliberations.

*My vision on ethics support has really widened because of this project. For me, CES was especially about moral case deliberations. That was the method, it's what I know, I am specialized in it […] but ethics support is of course more than moral case deliberations. (R7)*

##### Self-reflection on existing CES practices

Respondents stated that participating in the RQA project fostered self-reflection about their own CES practices. This reflection was stimulated particularly by preparing documentation for the visiting CES practitioners:

*This [self evaluation] is always useful in the sense that it makes you stop and think again, as in what are we actually doing in that area. And what is our position. And how is it conveyed, of course that was also asked by the Board of Directors. (R6)*

Or by performing the assessment of CES in another organization.

*Of course you're going to reflect on how they do it and how we do it. (R10)*

The final report written after the assessment also assisted CES practitioners in reflecting on their practices.

*What I found very confrontational was the culture at our institution. How can I put it, the culture is like a coat that you grab. You don't really know what it looks like but you grab it time and again. But the moment that someone starts describing your coat, you suddenly become aware of the coat you're wearing. And that is what they did. And in the process, they also indicated how loose and unstructured our organization was, compared to the institution they come from. To me, that was an eye-opener. (R7)*

#### The need for a learning community (NEON)

The findings show that all respondents were motivated to further professionalize CES services within their organizations and emphasized the need for a learning community centred on CES. Respondents aim to further professionalize their CES practices, for instance, by facilitating peer coaching groups. Moreover, the Dutch NEON CES network is regarded as supportive; it facilitates participants to meet fellow CES practitioners from other organizations who are working on similar objectives.

*I think it's a good thing that there is something like a national platform that deals very specifically with the development of ethics support. And I think that's the positive thing about NEON. […] For instance, what I think would be wonderful is if NEON was also facilitating towards members, in the form of professional associations, to give an example. Or … hmmm … or, you can also have a facilitating function in how you find each other. For peer review purposes, like that. (R10)*

#### Questions about the quality of the quality assessment

Respondents explicitly mentioned that they saw themselves as assessors who operate with an open attitude rather than as auditors assessing the quality of CES strictly following pre-formulated criteria. Although the open and dialogical learning approach regarding the quality of assessment created more room for open learning, it occasionally also seemed to limit the critical dimension of the RQA and the final reports. As one of the CES practitioners said about visiting another organization:

*Do not question too critically and systematically, instead think along with the objectives, as a critical, interested guest. (R7)*

This open appreciative attitude, rather than auditing (judging and scoring) CES practices, is what was also expected from assessors who visited another organization:

*That was also the reason that they wanted to be at all of our discussions. Because they seemed to be really afraid that things would come to the fore or would be included in the report. Things that could come back to bite them or something like that […] So in the end, after the discussion [with the host] we also changed some things in the report, at their request. We actually did that. Because in the end, we don't want to harm anyone, right? (R2)*

Protecting and respecting the privacy of fellow CES practitioners occasionally resulted in less critical reflections. One respondent even argued explicitly that every CES practitioner delivers good CES in his or her own way.

*There was someone who presented something different than the others. It is important to include that in the report. At the same time, you don't want that to be traced back, so how do you go about it? Yeah, okay, hmmm [silence]. Yes, I believe I'm cautious, in such a situation I think somehow or other you are also a guest of such an organization, and you cannot just rip through their rather delicate and vulnerable structures with what you think, like a bull in a china shop. (R4)*

Participants also mentioned that their background and competences, as well as their abilities to assess the quality of CES, influenced the quality of their assessments. Most respondents, having only been educated in a specific CES method (e.g., moral case deliberation), did not regard themselves to be all-around clinical ethicists or clinical ethics support experts.

*I feel quite competent as an assessor. But I do not feel like an ethicist. And, er, that also has to do with the fact that I [silence] have not followed a specific training for it. (R7)*

## Discussion

There are a variety of approaches to fostering the quality of (CES) services in healthcare organizations [20, 24, 26]. In this study, we evaluated the RQA project on the quality of CES in Dutch health care organizations. By connecting to actual CES experiences and practices, the aim of the responsive quality assessment project was to co-create an open learning process in which both the quality of CES and how to foster the quality of CES were reflected upon.

We observed that the motives of Dutch clinical ethics practitioners for participating in responsive quality assessments of their clinical ethics practices were quite straightforward. The practitioners demonstrated a learning attitude towards other practices, often, but not necessarily, with strategic goals in mind concerning further implementation of CES in their own organizations. Respondents had a desire to learn from others and expressed hope that, through participating in the assessment, their CES work will be acknowledged in their own organizations as relevant to fostering quality of care. The practitioners observed that CES often lacks a formal status within organizations, as well as a substantial budget and clear responsibilities and structure. Participation in the responsive quality assessments led to a number of changes regarding CES in Dutch health care organizations: acknowledgement of the relevance of CES; CES practices being formalized; the development of new CES-related activities; and increased reflection on existing CES practices. Respondents were motivated to further professionalize CES services and emphasized the need for a learning community through the Dutch network for CES (NEON). Finally, we found that within the quality assessment, the open attitude of participating CES practitioners, the willingness to protect (the privacy of) fellow CES practitioners, and the lack of sufficient experience to be a CES assessor sometimes seemed to result in less critical reflections during and after the assessment.

In this section, we will further discuss the interplay between the research context, the process and the perceived outcomes of the RQA project. We differentiate between the wider context of CES in Dutch health care organizations and the context of the RQA project itself. We conclude this section by discussing how, if worthy of further application, to improve this particular way of evaluating the quality of CES.

### The interrelatedness of the context of CES and the RQA

Literature on the quality of CES services includes a wide range of issues: discussions on competencies of CES practitioners, quality of training programmes or CES activities, quality standards, codes of conduct and accreditation [20, 25, 33]. Studies evaluating the quality of CES differentiate between elements such as quality of care, patient outcomes, knowledge and skills in ethics, satisfaction of stakeholders and resource use [1, 4, 34, 35]. Our findings show that in the RQA project, the focus of the participants was primarily on further implementation and procedures of CES activities. Very few respondents concentrated on the goals and content of particular CES activities for a critical assessment at the level of content of the CES activity itself (such as statements about the quality of ethics committee meetings or their policies, advice and letters, or the quality of arguments within a moral case deliberation).

Our respondents are still fighting for the implementation of CES, fighting to legitimize its existence, to gain support from upper management and to solidify CES services within health care organizations. This focus seems related to the fact that CES is still a relatively new field in the Netherlands. Moreover, within the Dutch context of health care organizations, CES seems to be increasingly assessed in terms of parameters that are related to the shift from public to private health care provision [36, 37]. Therefore, CES services must be accounted for in terms of efficiency and effectiveness. Consequently, one of the key questions is whether it is defensible to invest in and provide time-consuming CES services [38, 39]. While CES is reported to improve multidisciplinary team collaboration, working culture and quality of care [14, 34, 35, 38–40], accountability of CES services in terms of efficiency and effectiveness remains complex. With scarce and limited empirical evidence of a concrete impact of CES on the quality of patient care and existing evidence mostly based on self-reports, the question remains whether all kinds of CES impacts of can or should be measured [41].

The focus on legitimizing CES services explains the strategic motivation of CES practitioners who participated in the responsive quality assessment project. A positive report may lead to concrete changes. Our findings show that there were indeed some perceived positive changes regarding acknowledgement of the relevance of CES and formalization of CES services due to participating in the assessment. For instance, questions that CES practitioners from another organization put to upper management asking why CES services were not compensated led to policy changes. This strategic motivation appears to have had an impact on the attitude of the CES practitioners who participated in the responsive quality assessments. Having strategic goals can conflict with writing and receiving a critical quality assessment report. In the interviews and in some of the reports, we observed this conflict when having a critical attitude did not serve the strategic agenda of either the assessors or the assessed. In the interviews, participants stated that they sometimes deliberately chose to refrain from expressing critical (normative) quality judgements. This strategic attitude may have hampered the learning process within the RQA.

The interviews and reports show how the wider context of CES and the perceived quality of CES interact. On the one hand, there is the urgency to legitimize CES services in terms of effectiveness, and on the other hand, there is the need for structural preconditions, including financial compensation, to develop CES services in a professional way. Fostering the quality of CES is challenging when CES activities are not supported financially. Nevertheless, CES communities need to demonstrate the quality and effectiveness of their activities to achieve understanding and support. Our findings show that if formalizing and acknowledging CES services is such a relevant theme, a recommendation would be to urge boards of directors and management of health care organizations to provide CES practitioners with a solid place within their organizations and to encourage CES practitioners to engage in the process of fostering the quality of their CES services. CES practitioners can contribute to this process by demonstrating the relevance and benefits of CES.

### Balancing of power in the RQA project

The strength of RQA is the active participation of CES practitioners. Our findings show that the context of CES practitioners may also hamper the learning process. We discuss the concept of responsive evaluation and review the power balance between evaluators (researchers) and respondents (CES practitioners) within the project.

The concept of ‘responsive evaluation’ was originally developed to offer an alternative to ‘preordinate evaluation’, which was regarded as the dominant approach [42]. Abma [27, 43–46] further developed this concept and offered a social constructionist methodology. A key assumption within this methodology is that realities are socially constructed in social-relational processes. Accordingly, when we aim to foster the quality of CES services by means of a responsive quality assessment, it is important to evaluate this project’s own (socio-historical) contexts and focus on what the participants in the project themselves think to be important issues. Therefore, one of the strengths of responsive quality assessments in general and this project in particular was the active participation of CES stakeholders in the research process. Active participation can stimulate and support changes in the actual CES services existing within health care organizations. This process is referred to as ‘process use’ [27]. Our findings indicate that the participating CES practitioners were motivated to participate and were inspired to develop new CES-related activities; the assessment fostered reflection on existing CES practices and focused on issues important to the CES practitioners themselves. In this project, this focus shed light on the importance of acknowledgement and formalization of CES practices within health care organizations.

Within the theory of responsive evaluation, the ‘evaluator’ plays an important role [27]. This role includes being the facilitator, fostering interactions and dialogue between participants, and being a ‘Socratic guide’, probing ideas, truths, and certainties that have previously been taken for granted and imparting new meaning and perspectives to support stakeholders in the process of evaluation. According to Abma [45], the responsibility of the responsive researcher is not to delegate power to participants but to enhance the quality of the dialogical process between participants, both in terms of its meaningfulness and its relational quality [47]. Within a dialogue, it is not always easy to maintain a Socratic, critical attitude, asking critical questions and bringing in new perspectives; it may instead be tempting to prioritize friendly relationships. The role of the evaluator may be to ensure a critical attitude within the RQA.

As stated in the Methods section, the RQA started with a training day to build collaborative relationships among CES practitioners and between CES practitioners and the project’s (research) facilitators. Although it was called a ‘training day’, ideas about how to responsively evaluate each other’s practices were collaboratively established. The role of the research team was mostly limited to organizing the training day and the interim meeting that occurred after one-third of the visits had taken place. The research team offered a structure for conducting the assessments, but during the assessment process, the role of facilitator and Socratic guide was transferred to the CES practitioners themselves. The (research) facilitators were not present during the actual visits to the organizations or during the process of writing the reports. While this was mainly due to time and financial constraints, it was assumed that the participants, who were CES practitioners themselves, all had some experience with asking questions, probing ideas, truths, and certainties that have been taken for granted and imparting new meaning and perspectives. The findings show that participants mentioned that their abilities to assess the quality of CES (by asking critical questions) influenced the quality of the assessments. More guidance and support was needed from the research team to manage the political tensions that surround CES practices. Participants felt at risk of being disempowered by critical assessments, and assessors did not know how to adequately tackle these risks during visits and in writing their reports. During the interim meeting there was a call for more “servant leadership” by the (research) facilitators, which was provided after this meeting. For instance, the research team identified the needs of the participants and provided an example of a short version of a management report about the project. A more proactive role of the project team might have better supported less experienced individual participants during the assessments. This could have been achieved by contacting individual participants during the process of the RQA. The project team could also have encouraged the participating CES practitioners not only to describe features of CES activities and focus on implementation but also to draw their attention to the other elements of CES services. Or, at the very least, the project team could have helped participants differentiate the level and depth of the assessments.

## Conclusions

In the context of the various attempts to assess and foster the quality of CES services, responsive quality assessments can be viewed as a successful method that facilitates an open learning process by actively involving CES practitioners and their concrete practices. Responsive evaluation appears collaborative, participative and capable of generating change.

Our study contributes to the literature on how to assess and foster the quality of CES within health care. An RQA offers ample opportunities for dialogic, mutual learning on context-specific, practically relevant issues and, thereby, improvement of CES practices. Based on their participation in the RQA, respondents perceived a number of changes regarding CES in Dutch health care organizations: acknowledgement of the relevance of CES; CES practices being formalized; the development of new CES-related activities; and reflection on existing CES practices. The evaluation of the process of the RQA also made us aware that, to do an RQA well, it is important to provide a structure that enables differentiation between start-ups and well-developed CES practices. In the context of starting CES practices strategic motivations may hamper critical feedback between RQA participants. To overcome this, the RQA the research team could show “servant leadership”, train the competences of the assessors, and more intensively guide participants through the political arena that comes with assessing the quality of CES in health care organizations.

## Data Availability

The datasets used and analyzed during the current study are available from the corresponding author on reasonable request.

## Abbreviations

ASBH: American society for bioethics and humanities
CES: Clinical ethics support
ECQAT: Ethics consultation quality assessment tool
NEON: Dutch national learning network for ethics support
RQA: Responsive quality assessment
